# Reducing zoonotic and internal parasite burdens in pigs using a pig confinement system

**DOI:** 10.14202/vetworld.2017.1347-1352

**Published:** 2017-11-16

**Authors:** Kadek Karang Agustina, Ida Bagus Ngurah Swacita, Ida Bagus Made Oka, I Made Dwinata, Rebecca Justin Traub, Colin Cargill, I Made Damriyasa

**Affiliations:** 1 Department of Veterinary Public Health, Faculty of Veterinary Medicine Udayana University, PB. Sudirman St. Campus, Denpasar, Bali 80223, Indonesia; 2 Department of Parasitology, Center Studies on Animal Diseases Udayana University, Markisa Alleyway of Sesetan St. No. 8 Denpasar, Bali 80223, Indonesia; 3 Department of Parasitology, Faculty of Veterinary Science University of Melbourne, Melbourne, Victoria, Australia; 4 Department of Livestock Production, South Australian Research and Development Institute, Hartley Grove, Urrbrae SA 5064, Australia

**Keywords:** confinement, parasite, pig, system, zoonotic

## Abstract

**Aim:**

This study was designed to validate the effectiveness of the pig confinement system (PCS) in reducing the prevalence of zoonotic and internal parasite burdens in pigs.

**Materials and Methods:**

Ten PCS households were selected together with 10 households practising traditional scavenging systems. Five pigs were monitored per household every 3 months for 15 months and blood and feces collected. Pigs received a single dose of oxfendazole at 30 mg/kg at baseline. Qualitative fecal examinations for intestinal parasite stages were performed, and serum was tested for antibodies to cysticercus of *Taenia solium*, *Trichinella* spp., and *Toxoplasma gondii*.

**Results:**

Based on fecal examination, the prevalence of pigs positive for parasite eggs was reduced in PCS pigs over consecutive samplings (*Ascaris suum* [14.3% to 0%]*, Trichuris suis* [46.9% to 8.3%], *Strongyle*-type eggs [81.6% to 8.3%], *Physocephalus* spp. [6.1% to 0%], and *Metastrongylus apri* [20.8% to 0%]) compared with increases in the number of pigs positive for parasite eggs in non-PCS pigs (*T. suis* [20-61.5%], *Strongyle*-type [60.4-80.8%], *Physocephalus* spp. [8.3-15.4%], and *M. apri* [20.8-34.6%]) and little change in pigs positive for *A. suum* (18.8-19.2%). While the prevalence of pigs with antibodies against to cysticerci of *T. solium* reduced in PCS pigs from 18% to 14%, the prevalence in non-PCS pigs increased from 42% to 52%. Antibodies to *Trichinella* were not detected, but the prevalence of *T. gondii* antibodies increased from 6% to 10% in PCS pigs and from 7% to 24% in non-PCS pigs.

**Conclusion:**

These data demonstrate the potential of a PCS to reduce the prevalence of pigs infected with zoonotic and internal parasites and thus the risk to human and pig health.

## Introduction

Pigs are the most popular livestock husbanded in Papua and West Papua, Indonesia, and have traditional, religious, and economic value. People in these areas rarely have latrines and tend to defecate in their gardens or the areas around their house. Pigs are commonly housed in buildings next to the family kitchen and held during the morning and evening in a common space in the center of the family compound where they defecate in the same area as dogs and children [1]. This close association between pigs, children, and dogs enables cross-infection with a range of parasites to occur. Common zoonotic parasite diseases transmitted by pigs are *Taenia solium, Trichinellosis*, *Toxoplasmosis*, and *Ascariasis*, and all of which contribute deleteriously to human health [2-5]. Common internal parasites in pigs include *Ascaris suum, Trichuris suis, Strongyle-*type helminths (*Oesophagostomum dentatum, Hyostrongylus rubidus, Gnathostoma* spp., and *Globocephalus* spp.), *Physocephalus* ssp., and *Metastrongylus apri* [6,7].

Economic losses caused by internal parasites can be significant, but farmers may not realize it because symptoms tend to be subclinical and pigs may still look healthy [3,8]. Parasites can decrease endurance by absorbing essential nutrients and interfering with vital organs, making pigs more susceptible to various diseases. The availability of synthetic chemicals for eliminating internal parasites has grown rapidly, and vaccines for *Cysticercosis, Toxoplasmosis*, and *Trichinellosis* are available [9-11]. Unfortunately, for small-scale pig farmers, they can be expensive and difficult to obtain [12].

To help address these issues in a sustainable manner, a pig confinement system (PCS) that separates and isolates pigs from both human and dog feces was developed in the highlands of Papua between 2002 and 2007. The modified PCS was designed to provide pigs with optimum housing and small paddocks (*lalekens*) sown with high protein pasture. Pigs were given access to water at all times and housed overnight in pens divided into wet areas for eating and dry areas, covered with cut grass, for sleeping. During the day, the pigs were rotated through 8 small paddocks with access to high protein forage pastures. They were moved to a new paddock when 50% of the leaf material had been consumed. Pigs were held in a special “dunging area” for 30 min each morning to reduce reinfection rates and contamination of pastures with parasite eggs and larvae. When pigs managed in a PCS system were fed balanced diets-based sweet potato roots and vines, they grew significantly faster (190±23.5 g/day) than either confined pigs with no access to pasture (150±33.3 g/day) and/or pigs in “free range system” (48.2±13.1 g/day) system. When compared with pigs reared in the existing scavenger system and fed diets of raw SP leaves roots and vines, pigs managed in a PCS grew up to 10 times faster (250-300 g/day). Families using PCS models produced an average of 12 pigs/sow/year, compared with 5 in traditional systems, and recovered the cost of new facilities within 3 years [1,13].

While the productivity benefits and profitability of the PCS model have been demonstrated in the previous studies, its potential to reduce the prevalence of pigs infected with zoonotic and internal parasites has not been studied. Therefore, the aim of this research was to investigate the effect of the PCS model on the prevalence of zoonotic and internal parasites in local pigs in Papua and West Papua.

## Materials and Methods

### Ethical approval

This study has been approved by the Animal Rights and Ethical Use Committee of Udayana University.

### The study area

The study was conducted in the Baliem Valley, Papua Province, and the Arfak Villages of West Papua Province, Indonesia.

### PCS and non-PCS farmers

Ten households that had been converted from a traditional scavenger system of pig production to a PCS model (PCS households) over 12 months were selected for the study together with 10 households that continued to practice traditional scavenging systems (non-PCS). All pigs were treated with a single dose of oxfendazole at 30 mg/kg 3 months before commencement of sample collection [14]. PCS farmers were given assistance in constructing a confinement system, incorporating the principles, and underpinning the PCS and advised to feed diets with plant material that has demonstrated efficacy against internal parasites for the pigs: Papaya seeds and betel nut [12,15]. Non-PCS farmers were encouraged to continue to produce pigs in the traditional non-confined way. Five pigs were monitored from each household at 3-month intervals over a treatment period of 15 months.

### Sample collection

Individual fecal and blood samples were collected. Feces were collected from the rectum or off the ground only if fresh and preserved in sodium acetic formaldehyde (SAF). Serum samples were separated from each blood and stored at −20°C until analysis.

### Fecal examination

Fecal samples were qualitatively examined for intestinal parasite stages by the SAF concentration technique [16].

### Enzyme-linked immunosorbent assay (ELISA) for *T. solium, Trichinella* spp., and *T. gondii*

All serum samples were qualitatively screened for IgG antibodies to cysticerci of *T. solium*, *Trichinella* spp., and *T. gondii* using ELISA. IgG antibodies against *T. solium* cysticerci were analyzed using an ELISA test developed using crude antigen at Udayana University (pers. comm., Swacita, October 2012). IgG antibodies against *Trichinella* spp. and *T. gondii* were measured using a commercially available enzyme immunoassay kit (PIGTYPE^®^: Cellognost*-Toxoplasmosis H).

### Statistical analysis

The obtained results on prevalence and seroprevalence of parasite infections in pigs were encoded and recorded in an excel database analyzed by descriptive statistics survey and were performed using Epi info version 7.2 for determination of means, percentage, and standard deviation.

## Results

**Figure-1:**
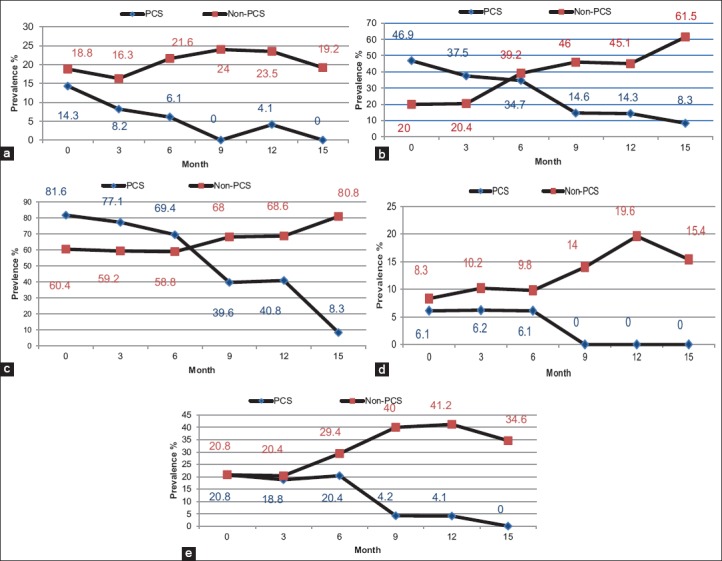
Prevalence of 5 parasite infections in confinement and non-confinement pigs during the monitoring period (0-15 months): (a) *Ascaris suum*; (b) *Trichuris suis*; (c) *Strongyle* type *helminths*; (d) *Physocephalus* spp.; (e) *Metastrongylus apri*.

The following gastrointestinal helminths were found in PCS and non-PCS pigs through qualitative fecal examination: *A. suum, T. suis, Strongyle*-type helminths, *Physocephalus* spp., and *M. apri*. The eggs of *A. suum* have been demonstrated to infect human as a zoonosis [17,18], and this species together with other helminths cause significant economic losses in pigs [3]. The prevalence of these helminth infections during the monitoring period are presented in Table-1 and Figure-1.

**Table-1:** Prevalence of parasites infection in confinement and nonconfinement pigs during the monitoring period.

Parasite	Period (month)	Mean (%)±SD	

		PCS	NonPCS
*A. suum*	0	14.3±0.350	18.8±0.388
	3	8.2±0.274	16.3±0.370
	6	6.1±0.240	21.6±0.412
	9	0±0	24±0.431
	12	4.1±0.274	23.5±0.424
	15	0±0	19.2±0.392
*T. suis*	0	46.9±0.499	20±0.404
	3	37.5±0.485	20.4±0.403
	6	34.7±0.476	39.2±0.488
	9	14.6±0.351	46±0.503
	12	14.3±0.350	45.1±0.498
	15	8.3±0.274	61.5±0.488
*Strongyle* type worm	0	81.6±0.387	60.4±0.491
	3	77.1±0.418	59.2±0.491
	6	69.4±0.461	58.8±0.492
	9	39.6±0.491	68±0.471
	12	40.8±0.491	68.6±0.464
	15	8.3±0.274	80.8±0.392
*Physocephalus* spp.	0	6.1±0.240	8.3±0.274
	3	6.2±0.240	10.2±0.303
	6	6.1±0.240	9.8±0.297
	9	0±0	14±0.351
	12	0±0	19.6±0.397
	15	0±0	15.4±0.359
*M. apri*	0	20.8±0.404	20.8±0.404
	3	18.8±0.388	20.4±0.403
	6	20.4±0.403	29.4±0.456
	9	4.2±0.198	40±0.495
	12	4.1±0.198	41.2±0.492
	15	0±0	34.6±0.476

*A. suum=Ascaris suum, T. suis=Trichuris suis, M. apri*=*Metastrongylus apri,* PCS=Pig confinement system, SD=Standard deviation

Figure-1 shows the overall prevalence of zoonotic and other internal parasites to decrease dramatically in PCS pigs during the treatment period (*A. suum* 14%±0.35 to 0% [a]*, T. suis* 49.9%±0.499 to 8.3%±0.274 [b], *Strongyle*-type helminths 81.6%±0.387 to 8.3%±0.274 [c], *Physocephalus* ssp. 6.1%±0.240 to 0% [d], and *M. apri* 20.8%±0.404 to 0% [e]), compared with an increase in the number of pigs positive for parasite eggs in non-PCS pigs (*T. suis* 20%±0.404 to 61.5%±0.488 [b], *Strongyle*-type helminths 60.4%±0.491 to 80.8%±0.392 [c], *Physocephalus* spp. 8.3%±0.274 to 15.4%±0.359 [d], and *M. apri* 20.8%±0.404 to 34.6%±0.476 [e]) and little change in pigs positive for *A. suum* (18.8%±0.388 to 19.2%±0.392 [a]).

**Figure-2:**
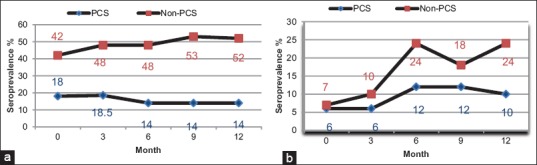
Seroprevalence of 2 parasite infections in confinement and non-confinement pigs during the monitoring period (0-12 months): (a) Cysticerci of *Taenia solium*; (b) *Toxoplasma gondii*.

Seroprevalence of parasite infections in confinement and non-confinement pigs are presented in Table-2 and Figure-2. The seroprevalence of pigs with antibodies against cysticerci of *T. solium* (Figure-2a) decreased in PCS pigs from 18%±0.388 to 14%±0.351, but the seroprevalence in non-PCS pigs increased from 42%±0.499 to 52%±0.505 after 12 months of treatment. Antibodies to *Trichinella* were not detected, but the seroprevalence of *T. gondii* antibodies (Figure-2b) increased from 6%±0.240 to 10%±0.303 in PCS pigs and from 7%±0.248 to 24%±0.505 in non-PCS pigs.

**Table-2:** Seroprevalence of parasite infections in confinement and nonconfinement pigs.

Parasite	Period (month)	Mean (%)±SD	

		PCS	NonPCS
*T. solium* cysticercosis	0	18±0.388	42±0.499
	3	18.5±0.381	48±0.505
	6	14±0.351	48±0.505
	9	14±0.351	53±0.499
	12	14±0.351	52±0.505
*T. gondii*	0	6±0.240	7±0.248
	3	6±0.240	10±0.404
	6	12±0.328	24±0.505
	9	12±0.328	18±0.485
	12	10±0.303	24±0.505

*T. solium=Taenia solium, T. gondii*=*Toxoplasma gondii,* PCS=Pig confinement system, SD=Standard deviation

## Discussion

The overall prevalence of zoonotic and internal parasites in pigs grown according to the PCS method shows downward trends compared to that of pigs from the traditional scavenging system (non-PCS), which shows rising trends. This is in agreement with the results of a previous study which supplemented feed using papaya seeds and betel nut [15]. These feed additions have thus been proven effective to reduce pig burdens of parasites. In the PCS method, pigs are confined in a specific area [1] and have limited access to the intermediate hosts of parasites and other parasite sources in the environment. This method is effective in protecting pigs from reinfection by parasites with indirect life cycles and pig-human associated parasites.

We found that the prevalence of *A. suum* decreased to 0% after 15 months PCS intervention. In endemic regions such as Guatemala and China, in which both *Ascaris* species (*A. lumbricoides* and *A. suum*) co-occur sympatrically, two host-associated transmission patterns are observed [19,20], with low levels of and gene flow between them. In areas of non-endemic human transmission, such as the United States, Denmark, and Japan, evidence indicates that *Ascaris* is a zoonosis and that pigs are reservoirs for human infection [4,17]. More recently, Zhou *et al*. utilized multilocus microsatellite genotyping to disapprove the former hypothesis by demonstrating that pig *Ascaris* can indeed serve as an important source of human *Ascaris* even in China, areas where the two coexist, similar to what we have observed in Papua [21].

Taeniosis and cysticercosis caused by *T. solium* are public health problems in many endemic countries where the persistence of this zoonosis is promoted by certain cultural, socioeconomic, and sanitary conditions [22]. Case of *cysticerci* of *T. solium* in pigs has a very close relationship with human defecation habits [23]. Pigs acquire infection through consumption of human feces or through feed and drinking water contaminated with *T. solium* eggs. In pigs, cysts commonly develop in the skeletal muscles, tongue, diaphragm, heart, and other organs, including the brain and eye [24]. Humans are then infected through the ingestion of raw or undercooked pork [5]. The PCS system breaks this infection cycle as demonstrated by our findings.

In this study, the seroprevalence of *T. gondii* rose slightly; a likely explanation is that pigs in the PCS system still have contact with cats and/or consume other potential food sources of infection. Cats play an important role in the spread of toxoplasmosis as they are the only species that excrete infective oocysts into the environment [25]. Pigs in confined pens still have a chance to become infected with *T. gondii*, because stray cats [26] and rodents [27] still have access, but this chance is lower than for scavenging pigs.

## Conclusion

These results demonstrate the potential of a PCS to reduce the prevalence of pigs infected with zoonotic and internal parasites and thus reduce the risk to human and pig health.

## Authors’ Contributions

KKA participated in the fieldwork, did fecal examination, analysis of data and manuscript drafting; IBNS carried out the laboratory work, ELISA test and participated in manuscript drafting; IBMO and IMD participated in the fieldwork, did fecal examination and participated in manuscript drafting; CC and IMD designed the research, arranged and control the PCS and non-PCS farmers, analyzed the data and participated in manuscript drafting; RJT designed the research, arranged and control the PCS and non-PCS farmers, analyzed the data and participated in manuscript drafting.
